# The Mechanism and Regulation of Disulfidptosis and Its Role in Disease

**DOI:** 10.3390/biomedicines14010228

**Published:** 2026-01-21

**Authors:** Yiming Wan, Mengjia Jing, Lumiao Zhang, Qianben Song, Xilin Ye, Zhenzhen Zhou, Wei Yan, Yu Fu

**Affiliations:** 1 Department of Gastroenterology, Tongji Hospital of Tongji Medical College, Huazhong University of Science and Technology, Wuhan 430030, China; hustwan123@163.com (Y.W.); jingmengjia@tjh.tjmu.edu.cn (M.J.); zhanglmtj@hust.edu.cn (L.Z.); songqianbenxy@163.com (Q.S.); 13359047902@163.com (X.Y.); 2 Department of Gastroenterology, Union Hospital of Tongji Medical College, Huazhong University of Science and Technology, Wuhan 430022, China; futureyu@hust.edu.cn

**Keywords:** disulfidptosis, cysteine, cystine, SLC7A11, NADPH, protein disulfide bonds, regulatory death, tumors, chronic diseases

## Abstract

Disulfidptosis is a recently identified form of regulatory cell death (RCD), Which has emerged as a research hotspot due to its distinctive feature of accumulating protein disulfide bonds, setting it apart from other RCD mechanisms. This discovery may offer new therapeutic strategies for cancer and various chronic diseases. This review aims to summarize the molecular mechanisms, inhibitors, regulatory networks, distinctions and connections between disulfidptosis and other regulatory death pathways, and the application of disulfidptosis in tumors and other chronic diseases. It also identifies unresolved issues and provides an outlook on future prospects.

## 1. Introduction

In the past, cell death was thought to be an uncontrolled and unregulated process. However, since Kerr et al. coined the term “apoptosis” in 1972, RCD emerged as a focal point in current research as a distinct category of cell death relative to ACD (accidental cell death) [1]. To date, many RCDs have been discovered, such as apoptosis, lysosome cell death, ferroptosis, cupric death, disulfidptosis, among others [2]. In their latest study, Liu et al. discovered that under glucose deprivation conditions, cancer cells that overexpress the cystine transporter SLC7A11, also called xCT, tend to gather excessive disulfide bonds, which triggers a unique type of cell death unlike traditional programmed cell death mechanisms like apoptosis or ferroptosis. This newly identified process has been dubbed disulfidptosis [3]. Regulatory cell death (RCD) often occurs due to complex signal transduction pathways, but the initial factors of different types of RCD vary, resulting in RCD with its own characteristics [4]. In Liu’s research, disulfidptosis was named for its characteristics of high disulfide bonds, such as the collapse of the actin cytoskeletal protein network disulfide bond stress and cystine overload [3].

In the literature on disulfidptosis, the collapse of F-actin is related to high expression of SLC7A11, indicating that SLC7A11 plays a central role in disulfidptosis [3]. SLC7A11 is a cysteine transporter. For every 1 mol glutamate released into extracellular space, 1 mol cysteine enters intracellular space [5]. SLC7A11 provides cysteine, an important raw material for the synthesis of intracellular GSH (glutathione). It has the capacity for intracellular cysteine synthesis, this being the rate-limiting factor for glutathione production [6]. Therefore, SLC7A11 enhances the survival ability of tumor cells in an oxidative stress environment [7]. As a result, SLC7A11 is often highly expressed in many tumor cells [8,9]. In addition, nicotinamide adenine dinucleotide phosphate (NADPH) is also an important participant in the formation of GSH. NADPH mainly originates from pentose phosphate pathway (PPP) and can reduce one molecule of cysteine transported into the cell into two molecules of cysteine for the synthesis of GSH [10]. Furthermore, tumor cells with high expression of SLC7A11 can increase PPP flux and upregulate PPP genes and GLUT1 (glucose transporter) expression, which may be a synergistic adaptation to the complex tumor microenvironment [11]. Therefore, when this synergistic relationship is broken and high SLC7A11-expressing tumor cells are exposed to glucose deprivation or GLUT1 inhibitor therapy, the tumor cells will rapidly undergo disulfidptosis [3].

**Figure 1:**
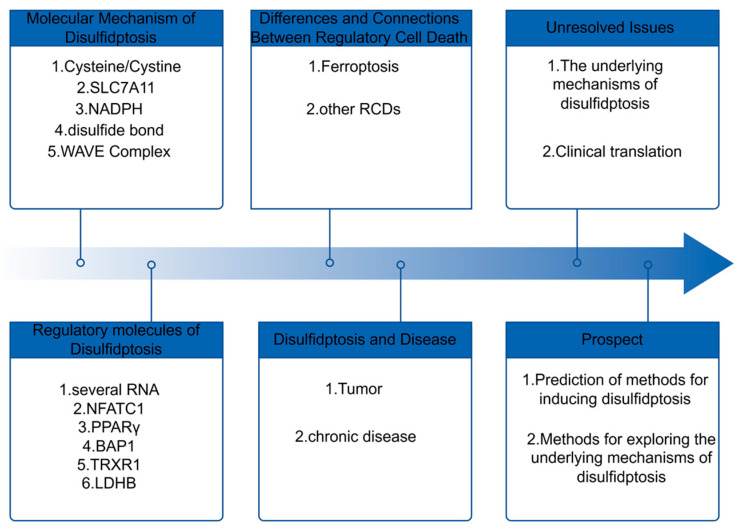
Overview of this Article.

Although the disulfidptosis mechanism remains unclear, recent research indicates its involvement in regulating and treating chronic illnesses such as renal cancer [3], pancreatic cancer [12], liver cancer [13], bladder cancer [14], non-alcoholic fatty liver disease [15], ulcerative colitis [16], hypertrophic cardiomyopathy [17] and other chronic diseases. Therefore, research on disulfidptosis is exciting for the future treatment of many diseases.To facilitate a clearer reading of the full text, we have drawn a flowchart of the entire narrative, as shown in Figure 1.

## 2. Molecular Mechanism of Disulfidptosis

In SLC7A11-overexpressing cells subjected to glucose scarcity, augmented cystine uptake exacerbates the NADPH deficit, resulting in reduced NADPH levels, GSH decline, cystine overload, abnormal disulfide bonds in actin cytoskeletal proteins, actin network collapse triggering cell contraction, morphological changes such as membrane blistering, and subsequent cell death. This entire process is called disulfidptosis [3].

### 2.1. Metabolism of Cystine/Cysteine

It is known that cysteine starvation induces ferroptosis [18]. In Liu’s study, even in low SLC7A11-expressing cells, disulfidptosis still occurred when excess cystine was added to the culture medium [3]. It can be seen that in ferroptosis and disulfidptosis, the content of cysteine plays an important role in both types of RCD. However, cystine possesses extremely low solubility, and its intracellular accumulation to high levels can exert significant toxicity [11], Cystine accumulation in cells and tissues results in multi-organ toxicity. For instance, cystine buildup in lysosomes of renal tissues gives rise to cystinosis, whereas its accumulation in other tissues can induce complications including portal hypertension, diabetes mellitus, photophobia, and thyroid dysfunction [19]. Furthermore, the transport of cystine via SLC7A11 is accompanied by excessive extracellular glutamate accumulation, which elicits neurotoxicity and causes peripheral neuronal damage [20]. Therefore, cells actually need an important non-essential amino acid—cysteine. Cysteine has numerous functions, such as participating in oxidative stress, metal binding, and transport [21], Moreover, this demand for cysteine is particularly strong in cancer cells. When cysteine cannot be synthesized, GSH is also depleted, results in lipid peroxide buildup, triggering ferroptosis [18], It also prevents disulfide bonds from being reduced, causing them to accumulate in large quantities and leading to disulfidptosis [3]. Thus, a more profound grasp of cysteine’s distinct metabolic processes will facilitate a more thorough examination into the roots of disulfidptosis.

Within cells, cysteine primarily has three destinations. The first destination is the synthesis of proteins and peptides, with protein synthesis being the primary destination for cysteine, accounting for a significant proportion of its utilization [21]. The second pathway involves taurine acting as a precursor in lipid metabolism, participating in the synthesis of cholesterol through a conjugation reaction to produce bile acids, and contributing to cellular energy metabolism. The third pathway involves cysteine synthesizing hydrogen sulfide (H2S), which is associated with certain cancers such as lung cancer [22].

Intracellular cysteine is primarily derived from three sources: SLC7A11-mediated uptake of extracellular cystine, the transsulfuration pathway (TSS), or glutathione degradation [23]. Since many cancers exhibit compromised TSS activity, they become dependent on SLC7A11 upregulation to satisfy cysteine demands. Consequently, SLC7A11 has emerged as a promising therapeutic target for inducing regulated cell death in cancer [24,25].

### 2.2. SLC7A11’s Regulation Is Important

In numerous studies on ferroptosis, SLC7A11 has been identified as an inhibitor of ferroptosis. Numerous studies have examined SLC7A11 cellular expression to determine whether tumor cells undergo ferroptosis, with the aim of treating tumors [26]. Enhanced SLC7A11 levels mitigate cellular vulnerability to ferroptosis, consequently safeguarding against cell death [27]. Research by Jiang, Zhang, Dixon, and colleagues demonstrates that SLC7A11 overexpression confers ferroptosis resistance in cancer cells [28,29,30]. Therefore, when drug resistance develops, relying solely on ferroptosis limits treatment options. It is known that glucose starvation induces apoptosis in cancer cells [31], In this case, disulfidptosis can be exploited to eliminate ferroptosis-resistant cancer cells. Furthermore, upregulating the level of SLC7A11 can enhance cellular sensitivity to glucose deprivation [32], Once glucose makes its way into the cell through specialized transporters, it quickly hops into the glycolytic pathway to form G6P. From there, G6P can keep progressing through glycolysis or, alternatively, be diverted into the pentose phosphate pathway under the influence of G6PD. This route is responsible for producing approximately 60% of the cell’s NADPH [33], thereby establishing a connection between SLC7A11 and the PPP and NADPH pathways. Therefore, similar to the regulation of SLC7A11 by ferroptosis, altering the expression level of SLC7A11 in a glucose-starved environment is a practical way to induce disulfidptosis.

### 2.3. NADP+/NADPH Ratio and Thiol-Dependent Antioxidant System

In the reduction of disulfide bonds, NADPH acts through the thiol-dependent antioxidant system, namely the thioredoxin system TrXRs and the glutathione-disulfide bond reduction system GSH/Grxs [34]. Therefore, changes in NADPH content are also important indicators in the occurrence of disulfidptosis.

The thioredoxin system (TrXRs) comprises NADPH, thioredoxin reductase (TrxR/TXNRD), and thioredoxin (Trx) [35]. The TrxR system can utilize the electrons donated by NADPH to reduce the disulfide bonds of substrates, and it can also cleave cystine into cysteine [36]. Furthermore, Trx is a repair protein that prevents abnormal disulfide bond formation in cells [37], Studies have found that inhibiting TrxR1 can increase the likelihood of disulfidptosis [38]. The electrons in the thioredoxin system can originate not only from NADPH but also from the GSH system, and they can work simultaneously and complement each other.

The glutathione-disulfide reduction system GSH/Grxs requires glutathione reductase Grxs, GSH, GSSG, and NADPH. Glutathione reductase uses the cysteine residue at its active site to bind with disulfide substrates to form mixed disulfides, then nucleophilically attacks the disulfide bonds of the substrates, reducing them to their thiol forms, while forming disulfide bonds itself. After the reaction, the N-terminus of glutathione peroxidase can bind with one molecule of GSH to form a mixed disulfide. The mixture is further reduced by GSH, reactivating the system while converting GSH to GSSG. Glutathione reductase then regenerates GSH from GSSG using NADPH [39]. The glutathione disulfide reduction system not only protects proteins from inactivation but also has a profound impact in the redox system [40].

Currently, reducing NADPH production or increasing NADPH consumption can affect the occurrence of disulfidptosis. Therefore, GLUT inhibitors, like glucose starvation, induce disulfidptosis by reducing NADPH production in the PPP, leading the accumulation of disulfide bonds. Xiang’s research team developed an innovative disulfidptosis-inducing artificial membrane system. This system simultaneously upregulates SLC7A11 protein expression in malignant cells, accelerating disulfide bond aggregation [41]. At the same time, intracellular reducing agents such as GSH and NADPH are overconsumed, increasing the NADP+/NADPH ratio, disulfidptosis thus ensues.

### 2.4. The Synthesis of Disulfide Linkages in Protein Structures

Disulfidptosis results from excessive disulfide bond buildup in cellular and structural proteins [3]. Many places house disulfide bonds, most notably within the endoplasmic reticulum, but also scattered across the Golgi apparatus and the intermembrane space of mitochondria. The creation of these protein disulfide bonds involves an oxidative process, which is vital in shaping and refining proteins as they fold [42,43]. To synthesize disulfide bonds within cells, disulfide bond exchange proteins or disulfide oxidases capable of de novo synthesis are required [44].

In the ER of mammalian cells, PDI is a key component in disulfide bond formation. Oxidized PDI can react with newly synthesized polypeptides to transfer disulfide bonds to them [45]. Disulfide bonds can form through several routes, but the main process hinges on the cooperative interaction between Ero-1 and FAD, which together facilitate the conversion of protein PDI into its oxidized form. Every disulfide bond created yields one molecule of H_2_O_2_ [46]. In addition, there is PRX4, which complements Ero-1. PRX4 is composed of five dimers [47], After sulfonylation, its conformation changes, and its cysteine residues form disulfide bonds with cysteine residues of adjacent peptides, serving as electron acceptors for the electrons generated by PDI oxidation [48], PRX4 uses hydrogen peroxide, a product of Ero-1, as its final electron acceptor to produce water, thereby preventing the accumulation of excessive hydrogen peroxide within ER. Another glutathione peroxidase, GPX7/8, forms disulfide bonds in a manner similar to PRX4, also utilizing hydrogen peroxide generated by the Ero-1 pathway to simultaneously form disulfide bonds [49]. Other potential disulfide bond formation pathways include VKOR, which can convert vitamin K epoxide into hydroquinone to generate disulfide bonds [50]. When Ero-1, PRX4, and VKOR are knocked down, cellular disulfide bonds decrease, leading to cell death [44]. Additionally, there are pathways independent of oxidative PDI, such as the quiescent enzyme QSOX, which can directly oxidize substrates to form disulfide bonds [45].

Ero1 and PDI are typically present in partially reduced forms in human cells, which appears to reflect the redox balance of the ER [51]. It is a common understanding that mistakes can pop up when disulfide bonds get oxidized, which might cause the creation of non-native disulfide bonds. To fix this, the endoplasmic reticulum needs a good supply of reducing agents to transform those non-native disulfide bonds back to the native ones, which maintaining proper protein folding and ensures they do their job as they should. Recent studies have shown that the reducing equivalents in the ER may originate from GSH in the cytoplasm [52], GSH maintains the PDI active sites in a reduced state, essential for the creation of native disulfide linkages [53,54], upon depletion of GSH, the rate of oxidative protein folding within the ER quickens, and the incidence of abnormal disulfide bridges formation rises notably [53,54,55]. Some studies have also suggested that cytoplasmic NADPH also acts as an electron donor [56]. NADPH is itself a donor for GSH production, so the occurrence of disulfidptosis may be related to the decline of NADPH and GSH, leading to massive production of non-natural disulfide bonds in the ER. The use of catalase and peroxidase can rescue disulfidptosis [57], The reason may also be related to the coordinated action between Ero-1, which produces hydrogen peroxide, and PRX4 and GPX7/8, which utilize hydrogen peroxide.

Unlike the ER, the Golgi apparatus does not contain numerous disulfide bond-related enzymes. Only QSOX1 is present, which can simultaneously perform the functions of EroI and PDI to form disulfide bonds [43]. In mitochondria, disulfide bonds are mediated by the thiol oxidase Erv1 and the redox-dependent receptor Mia 40 to meet the needs of oxidative respiratory chain [46].

### 2.5. Rac1 Activates the WAVE Complex (WRC) to Promote Arp2/3-Mediated Actin Polymerization and the Formation of Lamellar Pseudopodia

Disulfidptosis is not just about an excess of cystine leading to a buildup of disulfide bonds; it also features the creation of these bonds within cytoskeletal proteins, which results in the breakdown of the actin framework. In Western blot analyses, these proteins often display a tailing pattern, highlighting this distinctive characteristic of the process [3]. Regulation of actin involves WASP and WAVE 1, 2, and 3 [58], with the WAVE complex acting downstream of Rac1 [59]. Knockout of NCKAP1 (the most important part of the WAVE complex) or RPN1 in cells increases the resistance of cells to disulfidptosis [3]. However, knockout of NCKAP1 does not affect the expression levels of SLC7A11 or SLC2A3, cysteine uptake, or the NADP+/NADPH ratio, but only directly regulates disulfidptosis. It is known that Rac1 activates WRC to form pseudopodia [60], indicating that the Rac1-WRC-Arp2/3 pathway provides a target for disulfide bond overload in the actin network during disulfidptosis. It is known that knockout of NCKAP1 inhibits disulfidptosis. Zhong’s research revealed that NCKAP1 plays a crucial role as a tumor suppressor in hepatocellular carcinoma. The study demonstrated that this protein boosts Rb1/p53 pathway activation, thereby slowing cancer cell proliferation and improving clinical outcomes. Importantly, the findings establish NCKAP1’s mechanism of action—it controls the cell cycle in HCC lines specifically through targeted regulation of the Rb1/p53 axis [61].

**Figure 2:**
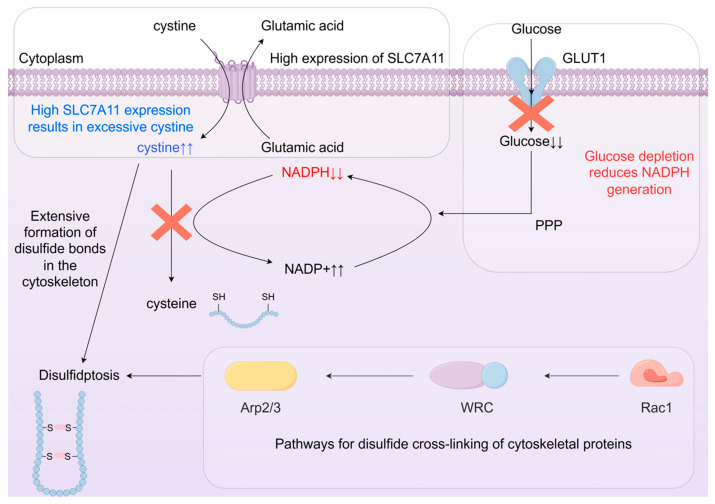
Mechanism of disulfidptosis [3]. Cells with high SLC7A11 expression undergo a decrease in intracellular glucose transfer under glucose starvation or GLUT inhibitor treatment, followed by a decrease in NADPH generated by the pentose phosphate pathway. The amount of NADPH is insufficient to completely reduce the cysteine transferred through SLC7A11, causing disulfide bond stress. abnormal disulfide cross-linking of cytoskeletal proteins, and ultimately causes cytoplasmic membrane detachment, leading to disulfidptosis. Among them, Rac1-WRC-Arp2/3-mediated lamellar pseudopodia play an important role in disulfidptosis.

Regarding the above mechanism, we have created a mechanism diagram of bisulfide death as shown in Figure 2.

## 3. Summary of Disulfidptosis-Related Inhibitors

Regarding bisulfide-induced death, we summarize some related inhibitors as shown in Table 1.

## 4. Regulatory Network of Disulfidptosis

Liu’s research revealed the process of disulfidptosis and its important nodes, such as SLC7A11, NADPH, cysteine metabolism, and glucose metabolism. At the same time, he successfully induced disulfidptosis in renal cancer cells using GLUT inhibitors, demonstrating the regulability of disulfidptosis [3]. Except GLUT inhibitors, numerous subsequent studies have revealed the regulation of disulfidptosis by RNA, proteins, and some pathways. These regulations are all carried out through the regulation of important nodes such as SLC7A11, glucose metabolism, or NADPH.

### 4.1. RNA Regulation of Disulfidptosis

#### 4.1.1. LncRNA

LncRNA constitutes a category of RNA molecules exceeding 200 nucleotides in length, which lack protein-coding capacity [62], LncRNA can bind to proteins, promote or inhibit their binding activity to the corresponding DNA sequences, and directly interact with DNA to silence genes, thereby regulating gene expression [63]. LncRNAs also contribute significantly to the differentiation, growth and death of cell, by binding to cell differentiation-related mRNA, or activating corresponding transcription factors (such as activating STAT3 to stimulate T cell activation) [63,64,65,66], and disulfidptosis is no exception.

In Sun’s study, he found that ALMS1-1T1 is a high-risk LncRNA in HNSCC [67]. It was also found that ALMS1-1T1 was co-expressed with SLC7A11, and after knocking down the ALMS1-1T1 gene, PPP-related genes were simultaneously downregulated, indicating that ALMS1-1T1 regulates the occurrence of disulfidptosis through PPP dependence under glucose starvation conditions. Western blot analysis of glucose-deprived HN-6 cells showed diffuse and high-molecular-weight bands corresponding to actin cytoskeletal proteins; this effect was amplified in ALMS1-1T1-overexpressing cells, indicating that ALMS1-1T1 promotes disulfide bond formation among these proteins. It is known that 2-DG, as an inhibitor of glycolysis, can directly produce NADPH through PPP to rescue disulfidptosis [68], and TCEP can produce a stronger disulfide bond reduction reaction to rescue cell contraction. Silencing ALMS1-1T1 resulted in a superior rescue effect of 2-DG compared to TCEP, demonstrating that the impact of ALMS1-1T1 knockout occurs through the NADPH pathway and not by directly influencing disulfide bonds [67]. However, it is worth mentioning that this study only analyzed the correlation between ALMS1-IT1 and SLC7A11 gene expression, and whether there is an interaction or synergistic relationship between the two in the occurrence of disulfidptosis has not been studied.

In Yin’s study, his team identify TMEM105 as a DRL in Pca. When TMEM105 was knocked down in a sugar-free environment, F-actin and cell contraction were remarkably enhanced, and the NADP+/NADPH ratio was enormously increased [69]. In further studies, Yin determined that TMEM105 regulates disulfidptosis by stabilizing β-catenin (an intracellular signal transduction protein that regulates cell proliferation and differentiation [70,71]) and enhancing the expression of c-myc (a transcription factor associated with glycolysis [72,73]) to induce GLUT1.

Moreover, Yao explored and uncovered increased levels of CASC8 in PDAC tumor samples [12]. CASC8 can influence cancer cell activity and may serve as a prognostic marker [74,75]. In Yao’s study, after treating cell lines with CASC8 knockdown with no sugar, a pronounced increment in cellular demise was noted, and this increase could be reversed by the disulfidptosis relievers TCEP and DTT, indicating that CASC8 has a disulfidptosis inhibitory effect. Yao’s KEGG enrichment analysis highlighted a notable disparity in metabolic pathways between the control and CASC8 knockdown groups, with glycolysis and the pentose phosphate pathway showing significantly higher activity in the former. Suppressing CASC8 expression led to a rise in the NADP^+^/NADPH ratio and a drop in glucose absorption, whereas overexpressing CASC8 produced the inverse effect. Subsequent GSEA by Yao uncovered a strong link between CASC8 levels and the expression of c-Myc target genes. Follow-up investigations confirmed a positive association between CASC8 and c-Myc, reinforcing their functional relationship [12].

#### 4.1.2. circRNA

CircRNA, like long LncRNA, was once believed to have no significant impact on cellular metabolism, proliferation, and differentiation. CircRNA was thought to merely accompany the gene splicing process [76,77,78]. As research has progressed, the roles of CircRNA in various cancers and regulatory pathways have begun to be uncovered [79]. For example, CircRNA can recruit RBPs to the promoters of oncogenes to activate gene expression, or inhibit promoters by competitively binding to transcription factor sites [80].

In Liu’s research, the expression of Let-7a-5p is downregulated by Circ_0022382, which consequently stifles the SLC7A11 expression by diminishing its mRNA levels. Knocking down Circ_0022382 results in a corresponding reduction in SLC7A11 levels, but it also leads to a substantial drop in NADPH levels and a subsequent decline in glucose metabolism. Notably, this decrease was not counteracted by ferroptosis inhibitors, hinting at a potential occurrence of disulfide stress [81], This seems to contradict common sense, but according to Yan’s conclusion, moderate levels of SLC7A11 can also induce disulfidptosis [7].

### 4.2. NFATC1 Induces Disulfidptosis by Sensitizing Osteoclasts to TXNRD1 Inhibitors Through Upregulation of SLC7A11 Expression

As mentioned earlier, NADPH can also inhibit disulfide bond formation through the thioredoxin system TrXR, and the most important and common inhibitory site in this system is TrxR1 [82]. TrxR1 can reduce cysteine to cystine [35].

In Zhong’s study, his team found that when T-cell nuclear factor (NFATC1) was upregulated, the transcriptional expression level of SLC7A11 was also upregulated. However, Zhong did not treat the osteoclasts with glucose starvation or GLUT1 inhibitors, but instead treated them with TXNRD1 inhibitors, which also led to F-Actin contraction and disulfidptosis [83]. NFATC1 sensitizes osteoclasts to TXNRD1 inhibitors by upregulating SLC7A11 expression, inducing disulfidptosis. Based on this, it can be inferred that certain enzymes in the thioredoxin system can also serve as targets for regulating disulfidptosis.

### 4.3. Inhibition of PPARγ Promotes Ferroptosis and Disulfidptosis

PPARγ is a ligand-dependent nuclear transcription factor that plays a significant role in tumor survival and development [84]. PPARγ activation governs glucose absorption and metabolic use in cells [85]. Therefore, PPARγ appears to have a similar effect to GLUT inhibitors.

In Zhang et al.’s study, when PPARγ was inhibited, markers of ferroptosis such as LPO were detected, indicating that cells underwent ferroptosis. However, the use of ferroptosis inhibitors could not completely inhibit cell death. Transcriptome sequencing indicated that PPARγ is related to glucose metabolism and upregulates SLC7A11 when inhibited. Zhang found that GLUT1 expression and NADPH oxidase were significantly reduced and cell death could be rescued by TCEP, and a tail band appeared in non-reducing gel electrophoresis [3], indicating that ferroptosis and disulfidptosis occurred simultaneously after PPARγ inhibition [86]. qPCR and Western blot analysis revealed that PPARγ inhibition increased HMOX1 expression, thereby inducing ferroptosis. After PPARγ was inhibited, SLC7A11 was also upregulated, promoting disulfidptosis in OSCC [86].

Zhang et al. combined research on ferroptosis and disulfidptosis, two oxidative stress-related death pathways, to show the possible synergistic effect of the two in causing regulatory cell death. However, in Zhang’s research, the cause of ferroptosis seems to be HMOX1 and its transcription factor NRF2, Therefore, in this case, SLC7A11 is no longer the protective umbrella of ferroptosis [86].

### 4.4. BAP1 Inhibits Disulfidptosis by Inhibiting SLC7A11-Mediated Cysteine Uptake and Reducing the NADP+/NADPH Ratio

BAP1 acts as a novel tumor suppressor through the deubiquitination of H2A monoubiquitination and subsequent regulation of gene transcription [87].

In Wang’s study, it was found that the expression level of BAP1 decreases as the expression level of SLC7A11 increases. In contrast to wild-type BAP1 cells, those overexpressing SLC7A11 exhibited more pronounced band tailing during non-reducing Western blot analysis following glucose deprivation. When SLC7A11 was subsequently suppressed, the occurrence of disulfidptosis in these cells was notably diminished. This suggests that BAP1 influences disulfidptosis through its regulation of SLC7A11. Later, two separate groups of cells were exposed to 2-deoxy-D-glucose (2-DG) under conditions lacking glucose. The results showed that, compared to the control, cells with elevated BAP1 levels had a less significant increase in the NADP+/NADPH ratio, implying that BAP1’s protective role against disulfidptosis is partly mediated by its impact on this redox couple [88].

### 4.5. Inhibition of TRXR1 Promotes Disulfidptosis Under Glucose Deprivation

Numerous studies indicate that suppressing TrxR1 expression can hinder cancer development.

In Tang’s study, it was found that activating TAZ and depriving glucose can promote the expression of TrxR1, where the increased expression of TrxR1 after glucose deprivation is an adaptive response. It is known that glucose deprivation induces cell apoptosis [89]. Tang found that inhibiting TrxR1 using AF or TRi-1 under glucose deprivation conditions significantly accelerated the rate of cell death, and demonstrated that this type of cell death could not be rescued by other RCD inhibitors such as ferroptosis. It can only be rescued by DTT and TECP, and characteristics of disulfidptosis, such as lamellar pseudopodia, were found in the actin cytoskeleton, indicating that inhibition of TrxR1 can promote disulfidptosis in glucose deprivation [38].

In addition, the study showed that when AF and glucose deprivation coexist, the occurrence of disulfidptosis does not depend on the high expression of SLC7A11. Therefore, Tang’s research has opened up a new path for the application of disulfidptosis.

### 4.6. STAT3 Inhibits G6PD Activity Through LDHB, Depletes NADPH, and Promotes Disulfidptosis in CD8+ T Cells

LDHB is primarily expressed in tissues with high oxidative metabolism, such as cardiac muscle and kidneys, and promotes the entry of lactate as an energy substrate into the tricarboxylic acid cycle. It plays a contradictory role in cancer, depending on the tumor type, and is also involved in the RCD process, nucleic acid synthesis, and lipid synthesis, among others [90]. STAT3 is a transcription activator that is overexpressed in tumors and plays an important role in tumor progression [91].

In Wan’s study, when LDHB was defective, G6PD activity increased, enhancing PPP flux and NADPH content, thereby inhibiting the occurrence of disulfidptosis. The authors also used bioinformatics technology to analyze that STAT3 binds to the promoter region of LDHB, promoting LDHB expression, thereby inhibiting G6PD activity, reducing NADPH, and promoting the occurrence of disulfidptosis [92].

According to Wan’s study, key enzymes in glucose metabolism can also affect the occurrence of disulfidptosis, proving that the induction of disulfidptosis is diverse and feasible, and providing a promising therapeutic strategy for targeting glucose metabolism-related enzymes to induce disulfidptosis.

### 4.7. Several Regulatory Pathways That May Be Related to Disulfidptosis

Most of the pathways mentioned above that are associated with disulfidptosis, such as the c-myc-GLUT1-pentose phosphate pathway axis [12,69], which is closely related to glucose metabolism [3,93]. Therefore, pathways related to sugar metabolism and important targets such as SLC7A11 are worthy of further study.

**Figure 3:**
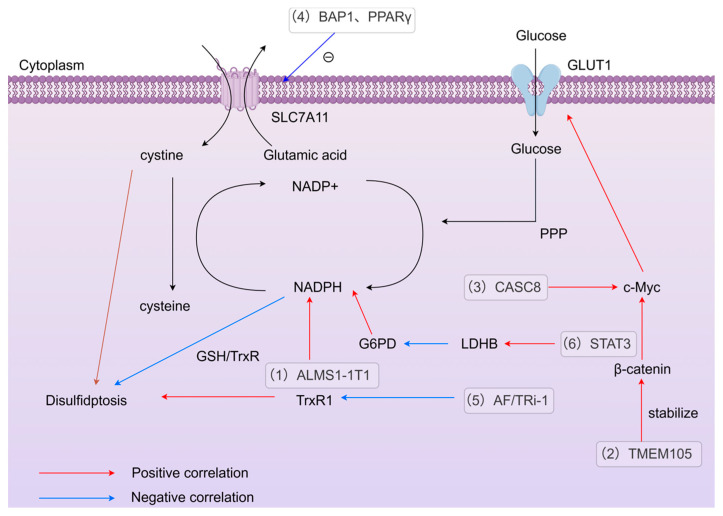
Molecules and pathways regulating disulfidptosis. (1) ALMS1-1T1 promotes NADPH production, and NADPH inhibits disulfidptosis through the reducing action of GSH/TrxR [67]. (2) TMEM105 induces GLUT expression and inhibits disulfidptosis by stabilizing the β-catenin-c-Myc axis [69]. (3) CASC8 promotes c-Myc-induced GLUT and inhibits disulfidptosis [12]. (4) BAP1 and PPARγ negatively regulate SLC7A11, reducing cystine transport and inhibiting disulfidptosis [38,86]. (5) AF/TRi-1 promotes cell death under glucose deprivation by inhibiting TrxR1 [83]. (6) STAT3 promotes LDHB expression to inhibit G6PD activity, thereby depleting NADPH and inducing disulfidptosis [90].

To summarize the above-mentioned regulatory mechanisms of disulfidptosis, we have created a regulatory network diagram of disulfidptosis, as shown in Figure 3.

## 5. Differences and Connections Between Regulatory Cell Death

Ferroptosis and disulfidptosis are not completely separate and can occur simultaneously [86]. Although different forms of regulatory cell death (such as disulfidptosis, ferroptosis, necrotic apoptosis, pyroptosis, and apoptosis) have significant differences in molecular mechanisms, triggering conditions, and morphological characteristics, these forms of RCD are all cross-regulated by redox homeostasis, metabolic reprogramming, and key molecules (such as p53 and the Bcl-2 family) and may act synergistically or antagonistically under pathological conditions. Therefore, elucidating their specificity and interaction networks can provide precise strategies for disease treatment targeting RCD.

### 5.1. Ferroptosis

Ferroptosis is an iron-dependent form of cellular demise characterized by the buildup of intracellular iron, disruption of the GPX4 antioxidant defense mechanism, and a surge in reactive ROS. These factors collectively drive excessive lipid peroxidation, causing catastrophic damage to cell membranes and culminating in the cell’s destruction [94].

On the surface, SLC7A11 inhibits ferroptosis while promoting disulfidptosis, appearing contradictory. However, closer analysis reveals this as a metabolic vulnerability arising from metabolic reprogramming in cancer cells. SLC7A11 inhibits ferroptosis by supplying cysteine, the precursor for GSH, and utilizing GSH as a cofactor for GPX4 to eliminate lipid peroxides. This process, however, consumes substantial NADPH to reduce cysteine. Under conditions of ample glucose, this system functions effectively. However, under glucose deprivation or NADPH depletion, this system collapses. The continuously supplied cysteine from overexpressed SLC7A11 cannot be adequately reduced due to insufficient NADPH, leading to massive accumulation of disulfide bonds—triggering disulfidptosis.

In the previous section, inhibiting PPAγ achieved the coexistence of disulfidptosis and ferroptosis. This seemingly contradictory phenomenon primarily arises from the upregulation of HMOX1, which leads to the accumulation of abundant free iron. This triggers the Fenton reaction, generating large quantities of hydroxyl radicals that directly drive lipid peroxidation. Concurrently, the upregulation of SLC7A11 fails to inhibit ferroptosis. Instead, it allows substantial amounts of cysteine to enter the cell, competing with GPX4 for NADPH. Consequently, the cell enters a state of metabolic collapse: NADPH depletion occurs, failing both to sustain the GPX4 system for ferroptosis and to reduce excessive cysteine, leading to disulfide bond accumulation and causing disulfidptosis. However, the deeper connection between the two types of cell death requires further investigation. Excitingly, In Li’s research, the team engineered a nanoferroptosis inducer that activates itself within the tumor microenvironment. This innovative construct features a polyethylene glycol-coated Fe-Bi-SS metal–organic framework loaded with high-Z elements, designed to enhance chemotherapy outcomes by specifically targeting ferroptosis pathways. They found that SLC7A11 can be upregulated adaptively. At this point, adding GOx to MOFs induces disulfidptosis, and the hydrogen peroxide produced by glucose oxidation can also undergo a Fenton reaction with ferrous ions to form highly oxidative hydroxyl radicals, thereby enhancing ferroptosis. This shows that disulfidptosis and ferroptosis can complement each other to improve therapeutic effects [95].

### 5.2. Other Regulatory Deaths

In addition to ferroptosis, other regulated cell deaths may also be associated with dithiothreitol-induced cell death, and we categorize them in Table 2.

Pyroptosis and disulfidptosis can work hand in hand to create a notable “synergistic” impact. In Zhu’s research, his squad harnessed GOx and SP as nano-inducers, labeling them as Pd2Sn@GOx-SP, to tweak intermetallic compounds. This clever maneuver sparked a potent anti-tumor immune reaction, thanks to the combined efforts of both pyroptosis and disulfidptosis [97].

Autophagy provides energy by breaking down cell components, which may alleviate the NADPH demand in disulfidptosis and thereby inhibit its occurrence [98]. The mitochondrial pathway of apoptosis may affect the NADPH/NADH balance. Furthermore, p53 promotes mitochondrial outer membrane permeability by regulating genes such as BAX/BAK in apoptosis, and some studies suggest that p53 may indirectly regulate disulfidptosis by regulating SLC7A11 expression, which may affect cysteine metabolism [99].

As an emerging form of regulatory cell death, disulfidptosis provides a multidimensional entry point for combined cancer therapy through its potential cross-regulation with apoptosis, necrotic apoptosis, and autophagy. In the future, it will be necessary to analyze the spatiotemporal dynamic interactions of death pathways in depth and develop combined therapies with controllable timing to ultimately achieve efficient, low-toxicity, and precise tumor elimination.

## 6. Disulfidptosis and Tumors

Disulfidptosis, can not only be used to establish prognostic models, but can also be induced in tumor cells as a form of tumor treatment.

### 6.1. Application of Disulfidptosis-Related Genes (DRGs) and Related LncRNA (DRL) in Tumors

Numerous studies have shown that discrepancies in the manifestation of genes or LncRNAs linked to disulfidptosis distinguish cancerous from healthy tissues. These disparities serve as valuable clues for identifying potential regulatory and therapeutic focal points, as well as novel biomarkers for tumor diagnosis. DPAG scores are assigned to DRGs in GC. APC11, a chosen DPAG in GC, serves as an independent prognostic marker and correlates strongly with unfavorable outcomes. APC11 is over-expressing in GC, and knocking out APC11 tremendously inhibits the proliferation and migration of GC cells [100], Similarly, Tensin4 (TNS4) in lung adenocarcinoma [101], ACTN4 and LCN2 in endometrial cancer [102], CCNA2 and RPN1 in hepatocellular carcinoma [103,104], INF2 and MEGF10 in osteosarcoma [105], CAPZB in diffuse large B-cell lymphoma [106], AC005840. 4 in bladder cancer, etc. Furthermore, the high-risk group identified using this DRL in bladder cancer showed higher sensitivity to sorafenib and oxaliplatin, demonstrating a link between disulfidptosis and existing anticancer drugs [107]. In particular, Zhang’s study found that inhibition of the disulfidptosis gene MYH9, which is highly expressed in liver cancer, is not only associated with low survival rates in liver cancer, but also with resistance to sorafenib, and that inhibition of MYH9 can reverse sorafenib resistance [108].

### 6.2. Inducing Disulfidptosis in Tumors to Achieve Therapeutic Effects

Combining disulfidptosis with ICIs has emerged as a novel approach [109]. In Du’s review, this approach is described as theoretically feasible, providing insights into the potential of combining the two for cancer treatment [109]. Recently, Shu’s study reveals the regulation of PD-L1 expression in glioblastoma cells by modulating the disulfidptosis-T cell exhaustion (Tex) pathway, indicating that the combination of the two has unlimited prospects for treatment [110].

Not only through the immune environment, some small molecule compounds can also induce disulfidptosis in tumors. In Shi’s research, they found that Gaudichaudione H (GH) is a natural compound, which was found in the application of hepatocellular carcinoma: GH is not only related to GSH and NADP+/NADPH metabolism, but also can upregulate SLC7A11 under glucose starvation conditions to induce disulfidptosis [13]. However, this raises some questions: How can GH be accurately localized and concentrated in in vivo experiments, especially in the microenvironment of liver cancer cells in humans? How is it degraded?

Previous research indicates that SDT (sonodynamic therapy) offers benefits like being minimally invasive and capable of deep tissue penetration, enabling effective treatment of deep-seated tumors [111]. In Wang’s latest study, nanotechnology was combined with ultrasound technology. The SPCP/CCP@Bay system consists of biodegradable photodynamic pseudoconiugate polymers (SPCP) and cysteine-containing polymers (CCP), loaded with Bay-876 (a GLUT inhibitor) [14]. This system effectively accumulates and releases cystine in bladder tumors, inhibiting GLUT and glucose metabolism and inducing disulfidptosis, while ultrasound can induce ICD and synergize with α-PD-1 to inhibit tumor.

## 7. The Potential Value and Application of Disulfidptosis in Chronic Diseases

Disulfidptosis is also associated with some chronic diseases and diseases related to immune dysregulation, such as osteoarthritis, intervertebral disc degeneration, pulmonary hypertension, heart failure, hypertrophic cardiomyopathy, gingivitis, and sepsis. DRGs have different degrees of differentiated expression in these diseases and are considered to be potential targets for targeted therapy. As precancerous lesions of colon adenocarcinoma, UC and NAFLD are now hot topics of research in relation to disulfidptosis.

### 7.1. Application of Disulfidptosis in NAFLD

In Luo’s study, it was found that DRGs were significantly elevated in NAFLD and were associated with immune cells such as regulatory T cells. MYL6 was identified as a contributor to NAFLD development and a promising future treatment target [15].

### 7.2. Application of Disulfidptosis in UC

Recent studies have elucidated the critical role of disulfidptosis in the pathogenesis and treatment of UC. Song et al. verified the existence of disulfidptosis in UC, observing that the upregulation of the disulfidptosis-promoting gene SLC7A11 and the downregulation of the inhibitor NUBPL significantly correlated with patient responses to TNF-α inhibitors [112]. Similarly, Yang et al. identified SLC3A2 as a central hub for disulfidptosis, finding that elevated expression of disulfidptosis-related genes (DRGs) predicted non-response to TNF-α inhibitors, whereas responders exhibited reduced DRG levels. Their work suggests disulfidptosis drives acute inflammation in UC, highlighting it as a promising therapeutic target [16]. Furthermore, Jiang et al. demonstrated that disulfidptosis-mediated regulatory cell death exacerbates inflammation and disease severity [113]. Most recently, investigations into the link between UC and colon adenocarcinoma identified CD2AP and MYH10 as key DRGs. Molecular docking analyses revealed that agents such as budesonide and sulfasalazine exhibit high binding affinity to these disulfidptosis targets, with simvastatin and phenytoin sodium also showing potential to modulate this pathway in both conditions [114].

## 8. Unresolved Issues in Disulfidptosis

The regulatory pathway of RCD is complex and intricate. Even though Liu’s research has revealed some of the conditions under which disulfidptosis occurs, the mechanism and regulation remain unclear and need to be further investigated.

It is well known that protein formation requires production and modification by the ER and Golgi apparatus, so disulfide bonds are also formed in the ER and Golgi apparatus, and some protein disulfide bonds even exist dynamically [115,116]. But why does the accumulation of disulfide bonds in disulfidptosis occur significantly in the cytoskeletal protein actin network? Current research has only focused on disulfide bonds between cytoskeletal proteins, and the formation of disulfide bonds in other regions has not been thoroughly investigated, which may involve other rapid signaling pathways.

Disulfidptosis correlates with oxidative stress, and ROS is significantly increased in cells that highly express SLC7A11 under glucose starvation [11]. However, different ROS scavengers show completely different effects on the rescue of disulfidptosis. Tempol and Trolox have negligible rescue effects, while catalase has a very significant rescue effect [117]. Mitochondria are essential for cellular disulfide bond formation. When the ROS produced by mitochondria decreases, the disulfide bonds of actin decrease, while the inhibition of peroxidase and catalase leads to an increase in the content of disulfide bonds [118]. Therefore, in disulfidptosis, the relationship between mitochondria, ROS (hydrogen peroxide), and disulfide bonds is worth further study.

## 9. The Prospect of Disulfidptosis

Liu’s research pioneered a brand new RCD, which is closely related to the basic characteristics of certain tumors or chronic diseases, providing markers, possible treatment strategies, and so on [119]. Subsequent studies have also provided many methods that can be learned to make the future of disulfidptosis traceable. This article also speculates on possible future research directions based on existing research.

### 9.1. Combined Use Inhibitors

In the above-mentioned induction pathways of disulfidptosis, each step has a key enzyme or molecular target, such as the expression of SLC7A11, glucose metabolism, the NADP+/NADPH ratio, TrxR, and GSH/Grxs, which can be regulated by inhibitors to regulate disulfidptosis. However, multiple inhibitors have not yet been applied simultaneously within the same cell or tissue. For example, the combination of GLUT1 inhibitors with TrXR system inhibitors may further enhance efficiency or provide additional targets for localization. Based on the previously elucidated mechanisms of disulfidptosis, NADPH emerges as a promising therapeutic target; however, achieving its precise depletion within target cells remains a challenge. Leveraging the aforementioned nanomaterial-based induction strategies, we propose targeting multiple disulfidptosis-related genes (DRGs), specifically SLC7A11. By engineering a nanoplatform that integrates NADPH-consuming agents (e.g., glucose oxidase [GOx]) with inhibitors of NADPH biosynthesis (such as those suppressing the pentose phosphate pathway or TCA cycle dehydrogenases), we aim to achieve precise NADPH exhaustion, thereby inducing disulfidptosis with enhanced efficacy.

### 9.2. Predicting Using Molecular Docking Between Disulfide Death-Related Genes and Potential Drugs

In Jiang’s study [16], the authors conducted enrichment analysis in ulcerative colitis to identify disulfidptosis genes that are highly expressed in UC, and used drugs such as budesonide and sulfasalazine to perform molecular docking with highly expressed genes, finding that the two have high affinity. This provides a new idea for discovering drugs that induce disulfidptosis.

Disulfidptosis is now an emerging field that deserves broader exploration. Research on disulfidptosis is also limited to important indicator targets such as SLC7A11 and NADPH. In the future, the scope of research on disulfidptosis can be expanded to include, for example, glutamate metabolism related to SLC7A11, sugar metabolism related to NADPH, the PPP, key enzymes in the tricarboxylic acid cycle, and so on. Therefore, exploring this novel form of cellular demise is truly fascinating.

## Figures and Tables

**Table 1:** Summary of Disulfidptosis-Related Inhibitors.

	Inhibitor Target	Inhibitor Name	Mechanism
TrxR system	Trx	1-Methylpropyl-2-imidazolidine disulfide (IV-2) (also known as PX-12)	By irreversibly binding to Trx1’s Cys73, it blocks TrxR1 substrate activity.
		2-Hydroxybenzamide isoxazole (SAHA)	SAHA enhances endogenous TBP-2 (a Trx inhibitor) expression, indirectly activating ASK1 to induce apoptosis.
	TrxR	Gold compounds	Targeted reduction forms of TrxR and mitochondria
		Arsenic trioxide (ATO or As_2_O_3_)	induces irreversible oxidation of TrxR.
		Nitroaromatic compounds (DNCB)	Definitively block TrxR through dinitrophenylation at the carboxyl-terminal motif’s cysteine and adjacent selenocysteine residues.
		Platinum compounds	Specific and irreversible inhibition of TrxR
		Polyphenolic compounds	Block the redox-active C-terminal site of TrxR
		Auranofin (AF)	Potent TrxR inhibitors
		TRi-1	Selective TrxR 1 inhibitors
	SLC7A11	Erastin and its derivatives	Inhibiting the expression of SLC7A11
		Sulfasalazine	
		Sorafenib	
Dithiol reducing agent	disulfide bond	DTT (dithiothreitol)	Directly targeting disulfide bonds, TCEP is more effective than DTT
		TCEP (tri(2-carboxyethyl)phosphine)	
Glutathione-disulfide bond reduction system	Glutathione reductase Grx	2-Acetamido-4-(4-(4-chlorophenyl)-1-piperazinyl)-6-(trifluoromethyl)pyrimidine	Selectively inhibiting human Grx1
		Curcumin	By covalently modifying the active site cysteine residue of Grx, its reducing activity is inhibited.
		Withaferin A	Downregulating Grx expression and inducing oxidative damage
		Auranofin	By binding to the active site -SH of Grx, inhibiting its function (while also inhibiting the targeted thioredoxin reductase TrxR)
	Glutathione reductase GR	Carmustine	Alkylating agent, irreversibly inhibiting its activity by modifying the active site histidine residue of GR.
		2-AAPA (2-acetamido-4-aminobenzoic acid)	Blocking the NADPH binding site
		Auranofin	Binding to the selenocysteine of GR, inhibiting electron transfer
		Sodium selenite	Inhibits GR activity by oxidizing its -SH group (to form selenium sulfide)
		Resveratrol	Downregulates GR expression and increases intracellular ROS levels
		Ellagic acid	Can inhibit GR enzyme activity
glucose metabolism	Glucose-6-phosphate dehydrogenase (G6PD)	2-DG	2-DG can theoretically inhibit G6PD to inhibit NADPH production, but in disulfidptosis it actually promotes NADPH production
		Dehydroepiandrosterone (DHEA)	Inhibits G6PD
	NADPH oxidase (NOX)	Diphenyl iodide (DPI)	DPI inhibits NOX activity by binding to the flavoprotein domain, reducing the oxidative consumption of NADPH
		VAS2870	VAS2870 inhibits enzyme activity by interfering with subunit assembly
	Malic acid enzyme (ME1)	N-acetyl-5-methoxytryptamine (NAT)	Malate dehydrogenase catalyzes malate decarboxylation to produce pyruvate, simultaneously generating NADPH; inhibiting ME1 reduces this pathway
	Isocitrate dehydrogenase IDH1/2	AG-120	Specifically targets IDH1 mutants to restore NADPH homeostasis

**Table 2:** The association between other RCD and disulfidptosis.

Type of Cell Death	Main Mechanism	Cellular Response	Connection with Disulfidptosis
Apoptosis	Caspase activation, DNA fragmentation	Cell volume reduction, membrane vesicle formation [96]	Both affected by oxidative stress; possible synergistic effect
Necroptosis	RIPK1/RIPK3 pathway, MLKL activation	Swelling, membrane rupture [96], inflammatory response [96]	Cell membrane rupture, possibly triggering an inflammatory response
Autophagy	Autophagosome formation and lysosomal degradation	Degradation of organelles and macromolecules [96]	Oxidative stress may lead to disulfidptosis
Pyroptosis	Inflammasome activation, Caspase-1	Cell membrane rupture, release of inflammatory factors [96]	Disulfidptosis may indirectly activate inflammatory response

## Data Availability

Data sharing is not applicable to this article as no new data were created or analyzed in this study.

## Abbreviations

The following abbreviations are used in this manuscript:

RCD	Regulatory cell death
ACD	Accidental cell death
SLC7A11	Glutathione transporter solute carrier family 7 member 11
GSH	Glutathione
NADPH	Nicotinamide adenine dinucleotide phosphate
PPP	Pentose phosphate pathway
GLUT1	Glucose Transporter 1
G6PD	glucose-6-phosphate dehydrogenase
PDI	protein disulfide isomerase
DRGs	Disulfidptosis-Related Gene
HNSCC	Head and Neck Squamous Cell Carcinoma
OSCC	Oral Squamous Cell Carcinoma
ROS	Reactive Oxygen Species
UC	Ulcerative Colitis
NAFLD	Non-Alcoholic Fatty Liver Disease
ER	Endoplasmic Reticulum
GOx	glucose oxidase
SP	soybean phospholipid
LDHB	lactate dehydrogenase B
BAP1	BRCA1-Associated Protein 1
CircRNA	Circular RNA
RBPs	RNA-binding proteins
LncRNA	Long non-coding RNA
Pca	pancreatic cancer
PDAC	Pancreatic Ductal Adenocarcinoma
WASP	Wiskott-Aldrich syndrome protein
Ero-1	endoplasmic reticulum thiol oxidase 1
FAD	flavin adenine dinucleotide
VKOR	vitamin K epoxide reductase
ASK1	apoptosis signal-regulating kinase 1
GSSG	glutathione disulfide
G6P	glucose-6-phosphate
SAM	S-adenosylmethionine
SAH	S-adenosylhomocysteine
Hcy	homocysteine
CBS	cystathionine β-synthase
ICIs	immune checkpoint inhibitors
